# The Association Between Emotion Recognition, Affective Empathy, and Structural Connectivity in Schizophrenia Patients

**DOI:** 10.3389/fpsyt.2022.910985

**Published:** 2022-06-15

**Authors:** Martijn G. J. C. Koevoets, Merel Prikken, Doesjka A. Hagenaar, René S. Kahn, Neeltje E. M. van Haren

**Affiliations:** ^1^Department of Psychiatry, University Medical Center Utrecht Brain Center, Utrecht University, Utrecht, Netherlands; ^2^Department of Child and Adolescent Psychiatry and Psychology, Erasmus Medical Center Sophia Children’s Hospital, Rotterdam, Netherlands; ^3^Department of Psychiatry, Icahn School of Medicine at Mount Sinai, New York, NY, United States

**Keywords:** schizophrenia, emotion recognition, affective empathy, structural connectivity, MRI

## Abstract

**Introduction:**

Emotion processing deficits often occur in patients with schizophrenia. We investigate whether patients and controls differ in the association between facial emotion recognition and experience of affective empathy and whether performance on these emotion processing domains differently relates to white matter connectivity.

**Materials and Methods:**

Forty-seven patients with schizophrenia and 47 controls performed an emotion recognition and affective empathy task. T1-weighted and diffusion-tensor images (DTI) of the brain were acquired. Using Tracula 5.3, ten fibers were reconstructed and fractional anisotropy (FA), mean diffusivity (MD), axial diffusivity (AD), and radial diffusivity (RD) were extracted. Groups were compared on task performance, white matter measures and their interactions using ANCOVAs. Correction for multiple comparisons was applied.

**Results:**

Patients scored lower on emotion recognition (*p* = 0.037) and reported higher levels of affective empathy (*p* < 0.001) than controls. Patients with poor emotion recognition (PT-low) experienced stronger affective empathy than patients with similar emotion recognition performance as controls (PT-normal; *p* = 0.011), who in turn reported stronger affective empathy than controls (*p* = 0.043). We found a significant interaction between emotion recognition, affective empathy and anterior thalamic radiation AD (*p* = 0.017, *d* = 0.43). *Post hoc* analyses revealed that the correlation between AD and empathy differed significantly between all groups (empathy/AD in PT-low < empathy/AD in PT-normal < empathy/AD in controls).

**Discussion:**

In patients with poor emotion recognition, the negative association between anterior thalamic radiation AD and affective empathy was stronger than in patients with normal emotion recognition capacity. Possibly, axonal damage in fronto-thalamic structural connections, as part of a larger frontotemporal network, underlies the association between poor emotion recognition and higher levels of affective empathy in schizophrenia patients.

## Introduction

Emotion processing impairments are particularly pronounced in patients with schizophrenia compared to patients with other disorders. Patients with schizophrenia perform worse than patients with bipolar disorder or depression, who themselves have a lower performance as compared with controls (1–3). Emotion perception impairments (e.g., recognition and discrimination of emotional expressions) are a robust finding in patients with schizophrenia (4–7). The severity of emotion perception impairments differs across emotional valence. Moderate to high effect sizes are reported with respect to recognizing negative expressions (mostly fear and sadness), while identification of positive expressions (e.g., happy and surprise) is usually not affected (8, 9). Importantly, poor performance on facial emotion recognition tasks has clinical relevance as it correlates with worse cognition and social functioning (10–13) and with more severe positive and negative symptoms (11).

It has been proposed that the perception of others’ emotion is causally related to feeling and cognitively understanding that emotion (14–16), suggesting that individuals who score high on emotion perception also score high on experiences of empathy. Focusing on these two aspects of emotion processing, we set out to investigate whether a deficit in recognizing emotional facial expressions is associated with the level of experienced emotions in reaction to emotions of another person, i.e., affective empathy. Affective empathy is a broad and complex construct (17, 18). We limit its definition to the strength of one’s emotional response to the emotional experiences or states of others. Like facial emotion recognition, affective empathy contributes to relationship maintaining behavior and is key in maintaining an adequate social interaction (19). A recent review of the literature reported a lower level of affective empathy (defined as empathic concern, i.e., the tendency to feel warmth, compassion, or concern for others) in patients with schizophrenia as compared with healthy controls, which was most pronounced when using performance-based assessments, as compared to self-report (20). Moreover, a review on the Interpersonal Reactivity Index [IRI; (21)], a self-report questionnaire, revealed lower levels of empathic concern, perspective taking, and fantasy in patients with schizophrenia, but more personal distress (i.e., the tendency to experience unpleasant emotions when witnessing others in a negative situation). The effect size was highest for the personal distress subscale [hedges *g* = −0.71; (22)].

These two social cognitive constructs, i.e., emotion recognition and affective empathy, are usually assessed differently. Where emotion recognition capacity is derived from a test and represents an ability (or maximum performance), affective empathy is often measured via self-report questionnaires [e.g., IRI; (21)] and represents one’s own reflection on typical behavior or a personality-like construct (23). Here, we set out to investigate their association by assessing both in one performance task.

We adapted a task which was previously developed to measure neural responses to basic emotions with fMRI (24) to capture both constructs in one task. Participants were first shown a picture of a person with an emotional facial expression. Each picture is an image taken from a film clip. After rating the emotional facial expression from the person on the picture, participants were shown the corresponding film clip of the person experiencing an emotional event. The facial expression (picture) and the emotional event (film) reflected the same emotion. After each film clip, participants were asked to evaluate to what extent they experienced a set of emotions related to the person undergoing emotional events in the film clip. For this, we used the emotions in the empathic concern and personal distress subscales of the IRI.

A possible explanation for schizophrenia patients’ problems in facial emotion recognition and affective empathy may be found in altered connectivity of the brain’s structural network. Indeed, the social brain hypothesis states that deficits in social cognition and social functioning arise as a consequence of structural impairments in connectivity of the social brain network (25). Specifically, prefrontal and temporo-parietal areas and their connecting fibers have been proposed to underlie emotion processing (26). Schizophrenia patients show abnormalities in structural connectivity in the emotion processing brain network with a decrease in fractional anisotropy (FA) in fibers connecting the prefrontal and temporal areas, i.e., uncinate fasciculus, cingulum bundle, and arcuate fasciculus (27–29).

Fractional anisotropy is a measure of directional preference of diffusion within a voxel, derived from a ratio of the principal diffusivities (Supplementary Figure 1, for formulas and visual representation). Most structural connectivity research in schizophrenia used FA, however, mean diffusivity (MD), axial diffusivity (AD), and radial diffusivity (RD) can also be extracted. MD is derived by averaging the three diffusion directions, AD is defined by diffusion parallel or along an axon, and RD is the diffusion perpendicular to an axon, i.e., myelin. In addition to white matter disruptions of lower FA, also higher MD and RD are commonly identified in patients with schizophrenia, predominantly in fronto-temporal, interhemispheric, and thalamo-cortical regions [e.g., (30, 31)]. Investigating these measures, in addition to FA, may provide insight into the neurobiology underlying poor emotion processing.

In the current study, we investigate whether patients with schizophrenia and healthy controls differ in the association between emotional face recognition and affective empathy and whether this is related to differences in structural connectivity. We hypothesize that poorer performance on emotion recognition is related to a lower tendency to experience empathic concern and an increased level of personal distress. In addition, we expect that FA is reduced while RD and MD are increased in patients with the lowest performance on emotion recognition and in those who score low on empathic concern and high on personal distress.

## Materials and Methods

### Study Design

This study is part of the Social Cognition and Imaging in Psychiatry II (SCIPII) project at the University Medical Center Utrecht (UMCU), Netherlands, which ran between March 2015 and August 2017. The total procedure lasted approximately 8 h (divided over two visits). Sixty-seven patients with a DSM-IV schizophrenia diagnosis and seventy healthy controls were included in this study. All participants were assessed by trained clinicians. Patients were recruited from the psychiatry department of the UMCU and from local mental health care institutions. Controls were recruited via an online recruitment website^1^ and from advertisements on notice boards. Inclusion criteria were an age between 18 and 50 years old, Dutch speaking, and premorbid IQ > 80 [estimated by the Dutch Adult Reading Test; (32)]. Exclusion criteria were drug- or alcohol abuse in the 6 months prior to testing, history of closed-head injury, neurological illness, endocrinological dysfunction, and/or chronic use of medication known to influence brain functioning (except psychotropic medication), having ferrous materials in or around the body, and having claustrophobia. For controls specifically, exclusion criteria were having (or having had) a psychiatric disorder and/or having first or second-degree family members with a psychiatric disorder with psychotic features. For patients only, having an acute psychotic episode at the moment of testing was an exclusion criterion. All participants signed informed consent. Participants were financially compensated for their participation. This study was approved by the UMCU’s Human Medical Ethics Commission.

### Participants

After quality control of the magnetic resonance imaging (MRI) data and a check on completeness of the task data, 47 patients with schizophrenia and 47 controls were included for statistical analysis.

### Task

**FIGURE 1:**
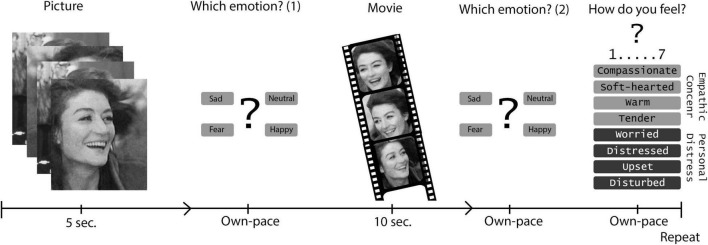
Emotion task: Emotion recognition and affective empath.

Emotion recognition and affective empathy were assessed in the same task [Figure 1; derived from (24)]. On a computer screen, participants were first shown a picture of a person with an emotional facial expression. Each picture is an image taken from a film clip. Participants were asked to choose one of four answer options (neutral, sad, happy, or fearful) that best matches the emotional expression portrayed in the picture. The total number of correct answer as well as the number of correct answers per emotion were counted. Subsequently, after each picture, participants were shown a film clip of the same person experiencing an emotion, i.e., the emotional content of the film clip matched the emotion of the previously shown facial expression. The film clip was silent and lasted for 10 s. After each film clip, participants were asked again to identify its emotional content (choose between neutral, sad, happy, or fearful). In addition, they were asked to evaluate to what extent they experienced specific emotions on two subscales, i.e., empathic concern and personal distress. The empathic concern subscale consists of positive emotions (warm, compassionate, soft-hearted, tender, and moved). The personal distress subscale entails negative emotions (worried, distressed, disturbed, upset, troubled, and agitated). Each emotion was scored on a 7-point Likert scale, ranging from 1 [not at all] to 7 [very strongly]. For each participant, the total score and mean score per subscale was calculated from their responses. In total, 30 pictures and film clips were shown. Due to technical problems, 4 patients and 6 controls could not complete the task.

### Magnetic Resonance Imaging Acquisition

Due to an MRI scanner switch halfway the project, scans were acquired on two 3 Tesla Philips Achieva scanners (Philips Medical Systems, Best, Netherlands), both using 8-channel SENSE head coils and running identical sequence parameters. Two transverse (anterior-posterior and posterior-anterior) diffusion weighted image (DWI) scans were made with the following settings: 30 non-collinear diffusion directions with b-factor = 1,000 s/mm^2^ and two diffusion-unweighted volumes with b-factor = 0 s/mm^2^; FOV = 240 × 150 mm, acquisition matrix resolution = 128 × 128, TE = 68 ms, TR = 7,178 ms, flip angle = 90°, parallel imaging SENSE factor = 2.5, 75 slices of 2 mm; no slice gap, reconstruction matrix = 128 × 128, no cardiac gating. The two DWI scans allowed correction for geometrical and intensity distortions caused by field imperfections (33). A whole head three-dimensional T1-weighted coronal spoiled-gradient echo scan was made with the following settings: FOV = 240 × 176 mm, acquisition matrix = 304 × 304, TE = 4.6 ms, TR = 10 ms, flip angle = 8°, 180 slices; no slice gap, 0.75 × 0.75 × 0.8 mm voxels, parallel imaging SENSE-factor = 2.5.

### Magnetic Resonance Imaging Processing

First, segmentation of anatomical T1 scans was done with FreeSurfer 5.3.0^2^. Briefly, T1-weighted image processing included automated Talairach transformation, intensity normalization, removal of non-brain tissue, segmentation of subcortical white matter and gray matter, tessellation of the gray/white matter boundary, and automated topology correction (34–38). Next, the surface was inflated for registration to a spherical atlas, utilizing an individuals’ cortical folding pattern to match cortical geometry across subjects.

Pial surface segmentations were visually checked and, when needed, manually corrected for mislabeling of tissue prior to analysis. Finally, an outlier analysis per Region of Interest (ROI) was done to indicate further visual checking for possible mislabeling. If more than 33% of ROIs exceeded 2 SD from the ROI mean [intracranial volume (ICV) corrected] the participant was excluded from the analyses (i.e., 5 patients and 2 controls).

Secondly, the two DTI scans were realigned, merged and corrected for possible gradient induced distortions using FSL’s TOPUP tool (39). Next, TRACULA corrected for eddy currents and simple head motion movement (40). A brain mask was created from the B0 diffusion images for rigid intra-subject registration with bbregister to the T1, which combined with the previously obtained T1-to-template (MNI152) registration results in the inter-subject registration. Next, brain masks were created from the T1 and expanded 2 mm into the white matter. They were subsequently transformed to individual diffusion space. After tensor fitting, all scalar output volumes were transformed to template space. Pathway priors were extracted from individuals’ brain masks and the *a priori* atlas data in template space, marking pathway intersects and neighbors for fiber tracking. FSL’s bedpostx fits the ball-and-stick diffusion model, which is fed to the Markov chain Monte Carlo (MCMC) algorithm. In combination with the pathway priors, it estimates the *a posteriori* probability distribution of the location of each pathway, giving the following diffusion measures: fractional anisotropy (FA), mean diffusivity (MD), axial diffusivity (AD), and radial diffusivity (RD) (Supplementary Figure 1). The mean of the entire path distribution as well as “along-the-tract” measures are given for 18 pathways (or fiber tracts); the corpus callosum (forceps minor and major), bilateral corticospinal tract, inferior longitudinal fasciculus, uncinate fasciculus, anterior thalamic tract, cingulum-cingulate tract, cingulum-angular tract, superior longitudinal fasciculus-parietal tract, and superior longitudinal fasciculus-temporal tract. Analyses were done on the mean of left and right tracts, resulting in a total of 10 tracts.

Scans were assessed on head movement, based on volume-by-volume translation and rotation as well as percentage of slice signal drop-out and signal drop-out severity. Participants with head movement measures exceeding 2 SD from the mean were excluded, which led to the additional exclusion of 4 patients and 4 controls. In addition, the quality of all tract reconstructions was visually checked. When a tract was only partially or not reconstructed, the number of control points were increased, and path reconstruction reinitialized. When a tract remained un-reconstructed, the participant was excluded (1 control).

### Statistics

Analyses were performed using Project R 3.2.3^3^ (see Supplementary Material for list of libraries). Data was tested for distribution normality with Kolmogorov–Smirnov’s tests. First, group differences between patients and controls on demographic variables were analyzed with a *t*-test (for continuous variables), chi-square test (for categorical measures), or a Mann–Whitney-*U* test (if distributions were skewed). Also, the included patients and controls were compared with those who were not included in this study on relevant demographic and clinical variables to test for attrition bias. Finally, white matter measures were compared between the two MRI scanners.

### Main Effects of Group

The distribution of the emotion recognition scores was highly skewed. Therefore, chi-square analyses were used to compare groups. Group differences between patients and controls on affective empathy were analyzed with a *t*-test. To quantify abnormalities in structural connectivity in schizophrenia patients, analyses of covariance (ANCOVAs) were used to compare average diffusivity measures for each fiber tract between patients and controls. Age, sex, and a dummy code for scanner were used as covariates. Effects sizes were estimated, i.e., Cramer’s *V* for non-parametric testing and Cohen’s *D* for parametric tests.

### Association Between Emotion Processing Measures

**FIGURE 2:**
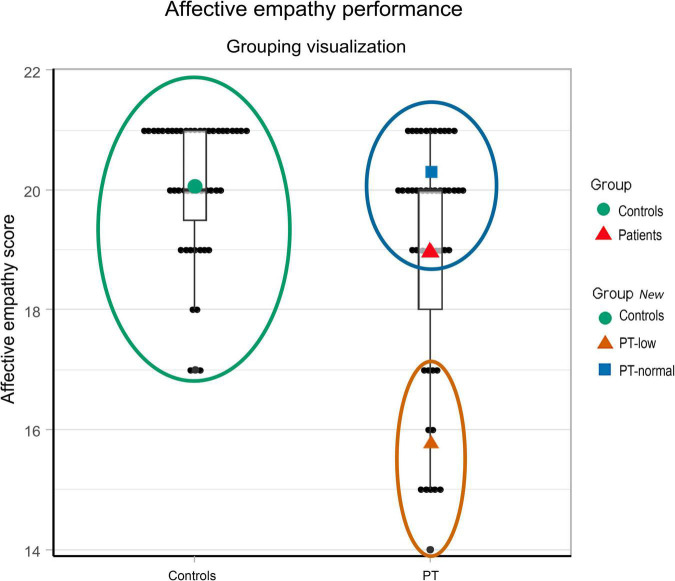
Performance on the affective empathy task in patients with schizophrenia and controls. Black dots represent individual scores. The green circle defines the control group with the green dot indicating the mean performance. The red triangle gives the mean affective empathy score of the patients, which was significantly lower than in controls (*p* < 0.001). For further analyses, we defined two groups of patients based on their performance on the emotion recognition task, i.e., those patients who performed in the same range as controls (PT-normal, represented in the blue circle) versus those scoring equal or lower than the control participant with the lowest score (PT-low, shown in the orange circle).

In controls, a task ceiling effect was found for emotion recognition, as almost all controls reached a perfect score. The distribution in patients was skewed. Therefore, we choose to define two groups of patients based on their performance on the emotional face recognition task, i.e., patients who performed in the same range as controls (PT-normal) versus patients scoring equal or lower than the control participant with the lowest score (PT-low) (Figure 2). To investigate the association between emotion recognition performance and reported affective empathy we compared levels of affective empathy between PT-normal, PT-low, and controls using ANOVA.

### Association Between Emotion Processing Measures and Structural Connectivity

First, the relationship between emotion recognition and diffusivity measures was investigated by comparing PT-normal, PT-low, and controls using ANCOVA. *Post hoc*, pairwise comparisons were done in case of significant main effects of group. Next, the relationship between affective empathy and the diffusion measures was compared between patients and controls using ANCOVA.

Finally, in our main analysis, we determined if the association between structural connectivity and level of affective empathy is dependent on performance on emotion recognition. Using ANCOVA, the association between level of affective empathy and structural connectivity was compared between PT-normal, PT-low, and controls. In all analyses age, sex, and scanner were added as covariates.

For all analyses, the False Discovery Rate (FDR) thresholding procedure was used to correct *p*-values for multiple comparisons. Analyses were repeated after adding IQ as a covariate.

## Results

### Demographics

Table 1 describes demographic and clinical information of the groups. Patients and controls did not differ significantly on age, sex distribution, own and parental level of education. Patients have a significantly lower premorbid IQ than controls (*p* = 0.005). Supplementary Table 1 describes the comparison between the included participants and those who were excluded based on poor image quality or missing data. There were no significant differences between included and excluded participants on demographic or clinical variables. Supplementary Table 2 describes the difference in white matter measures between MRI scanners. No significant differences were found.

**TABLE 1:** Information on demographic, clinical information and emotion processing performance for patients with schizophrenia and controls.

	Controls (*n* = 47)	Patients (*n* = 47)	Statistics	*p*	
	Mean (SD) or *N* (%)	Mean (SD) or *N* (%)			
Age (years)	32.88 (7.91)	35.88 (8.24)	*t* = −1.71	0.090	
Range	19.08–48.75	22.17–50.92			
Sex (N: M/F and F%)	43/4–8.5%	40/7–14.9%	χ^2^ (2) = 0.41	0.521	
Subject education (years)	15.8 (1.9)	16.1 (1.5)	*t* = −0.96	0.342	
Parental education (years)	13.6 (3.3)	14.2 (4.4)	*t* = −0.67	0.507	
Premorbid IQ	103.65 (7.46)	98.74 (8.84)	*t* = 2.88	0.005*	
PANSS total		52.13 (11.06)			
Positive		13.28 (3.90)			
Negative		13.49 (4.99)			
General		25.36 (5.59)			
MRI scanner (N: A/B and B%)	31/16–34.0%	35/12–32.4%	χ^2^ (2) = 0.46	0.499	
Illness duration (years)		14.10 (7.98)			
Antipsychotic medication Type [*N* (%)]					
Typical		2 (4.3)			
Atypical		40 (85.1)			
Both		1 (2.1)			
No medication		4 (8.5)			
Emotional face recognition – from picture	% Correct (SD)	% Correct (SD)			Cramer’s *V*
Happy	100.0 (0.0)	95.7 (14.2)	χ^2^ (4) = 8.74	0.033*	0.61
Neutral	91.5 (10.3)	85.8 (20.3)	χ^2^ (4) = 8.29	0.141	0.59
Sad	56.2 (10.0)	53.6 (12.6)	χ^2^ (4) = 3.54	0.316	0.39
Fear	96.2 (9.3)	89.7 (11.3)	χ^2^ (4) = 12.80	0.002*	0.74
Total	83.6 (5.0)	79.0 (8.6)	χ^2^ (4) = 14.96	0.037*	0.80
Affective Empathy – from movie clip					Cohen’s *d*
Empathic concern	2.15 (0.76)	2.69 (1.11)	*t* = −2.75	0.007*	0.57
Personal distress	1.55 (0.50)	2.19 (0.87)	*t* = −4.38	<0.001*	0.91
Total	1.78 (0.55)	2.36 (0.92)	*t* = −3.72	<0.001*	0.76

*Effect sizes for emotion recognition performance are given in Cramer’s V. Effect sizes for reported affective empathy are expressed as Cohen’s d. Parental education is mean of the number of years of formal education of both parents.*

*Significant at p < 0.05.

### Main Effects of Group

Patients performed significantly lower on the recognition of fear (*p* = 0.002) and happy (*p* = 0.033) faces and had a significantly lower total emotion recognition score (*p* = 0.037), with small to moderate effect sizes (Table 1). Note that controls made no errors in recognizing happy faces, hence mean (SD) = 6.00 (0.00). After viewing the movie, both groups showed a significant improvement in emotion recognition performance on the total score and all individual emotions, except for happy (Supplementary Table 3).

Schizophrenia patients reported significantly higher levels of empathic concern (*p* = 0.007) and personal distress (*p* < 0.001) after watching the movie clips than controls. Effect sizes were moderate to large (Table 1). Empathic concern and personal distress were highly correlated in both groups (patients: Pearson *r* = 0.78, *p* < 0.001; controls: *r* = 0.89, *p* < 0.001; Supplementary Figure 2) and showed high internal consistency (Cronbach’s α = 0.93 and 0.84). Therefore, we used the mean of all items as a measure of affective empathy (41).

Finally, no significant differences were found in the diffusion measures between patients and controls in any of the fibers (Supplementary Table 4).

### Association Between Emotion Processing Measures

To investigate the association between facial emotion recognition and experienced affective empathy, we compared affective empathy between PT-low, PT-normal, and controls. First, Supplementary Table 5 provides information on differences between PT-low and PT-normal on demographic and clinical variables. PT-low had significantly fewer years of education (*p* = 0.010) and a higher PANSS-total symptoms (*p* = 0.019) and PANSS-negative symptoms score (*p* = 0.005).

**FIGURE 3:**
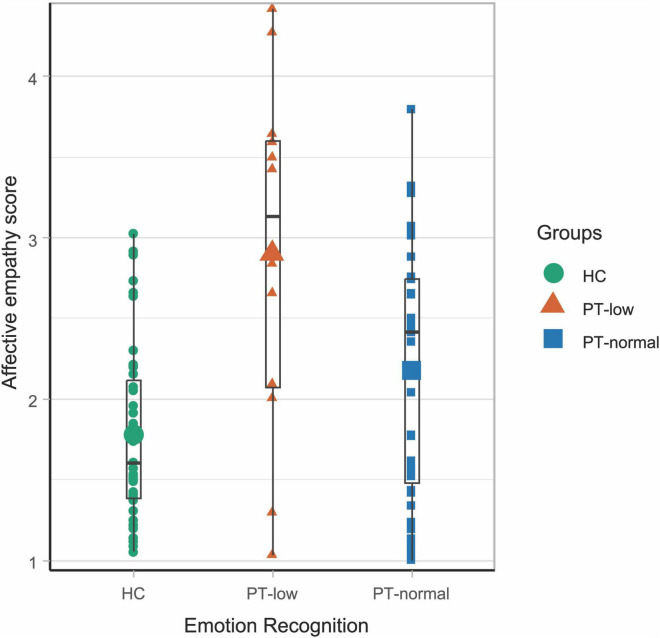
Performance on affective empathy task in controls and in the two patient groups (PT-low and PT-normal). The boxplot shows range, first and third quartile and median (black horizontal line). The larger symbols show the means; The green circles represent the controls (mean ± SD = 1.77 ± 0.55), the orange triangles represent patients with scores lower than controls (PT-low: mean ± SD = 2.90 ± 1.11) and the blue squares represent patients with scores in the same range as controls (PT-normal: mean ± SD = 2.2 ± 0.78).

A significant main effect of group was found for affective empathy [*F*(1,92) = 11.97, *p* < 0.001]. All pairwise comparisons were significant, with controls reporting lower affective empathy than PT-low (*p* < 0.001) and PT-normal (*p* = 0.044) and PT-normal scoring below PT-low (*p* = 0.010, Figure 3 and Supplementary Table 6). The pattern of findings was similar when we repeated the analysis in the individual subscales of affective empathy (i.e., empathic concern and personal distress) and for each emotion (Supplementary Table 6).

### Association Between Emotion Processing Measures and Structural Connectivity

No significant differences were found between PT-low, PT-normal, and controls in FA, MD, RA nor AD of the different fibers (Supplementary Table 7).

**FIGURE 4:**
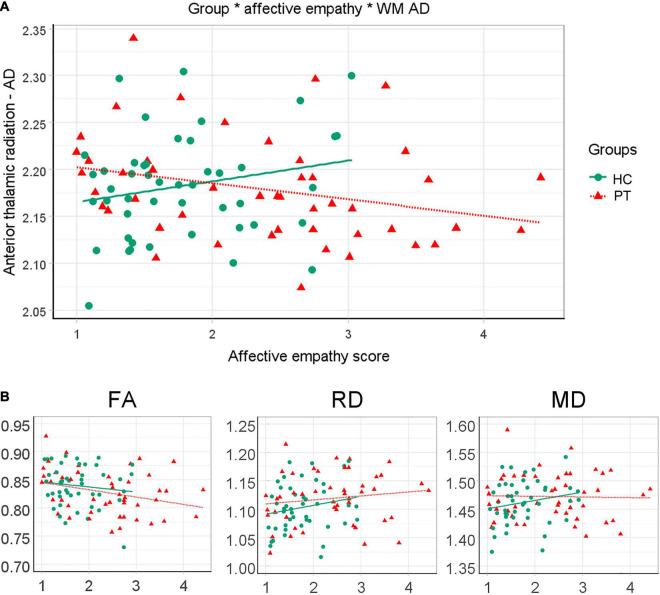
Differential relation between affective empathy and white matter diffusivity measures between patients and controls. **(A)** Anterior thalamic radiation AD*1000 is plotted against mean affective empathy in healthy controls (green circles/solid line) and patients (red triangles/small-dashed line). Dots are raw values lines are linear fitted trend lines. **(B)** Similar plots but for FA, RD*1000 and MD*1000 in the anterior thalamic radiation.

A significant interaction effect was found between level of experienced affective empathy and AD in the anterior thalamic radiation between controls and patients (Figure 4 and Table 2). That is, there is a negative association between AD in the anterior thalamic radiation and level of experienced empathy in patients while this association is positive in controls. The other diffusivity measures in the anterior thalamic radiation showed no significant interaction effect (Table 2). Findings were similar for the affective empathy subscales (Supplementary Table 8).

**TABLE 2:** Information on the association between affective empathy and measures of white matter diffusivity for **(A)** patients with schizophrenia and controls and for **(B)** patients with lower performance on emotion recognition, patients with normal emotion recognition and controls.

	*Means (SD)*						*Statistics*		
	Controls		Patients				*F*	*p* (adj.)	Cohen’s *d*
**(A)** Affective empathy									
Anterior thalamic radiation FA	0.834	(0.055)	0.828		(0.040)		*F*(1,92) = 1.06	0.349	0.21
Anterior thalamic radiation RD	1.113	(0.080)	1.119		(0.045)		*F*(1,92) = 0.01	1.077	0.02
Anterior thalamic radiation MD	1.470	(0.064)	1.472		(0.040)		*F*(1,92) = 1.44	0.267	0.25
Anterior thalamic radiation AD	2.182	(0.056)	2.179		(0.056)		*F*(1,92) = 6.04	0.018*	0.51
**(B)** Interaction (affective empathy and emotion recognition)			**PT-normal**		**PT-low**				
Anterior thalamic radiation FA	0.834	(0.055)	0.831	(0.041)	0.817	(0.035)	*F*(2,92) = 1.21	0.345	0.23
Anterior thalamic radiation RD	1.113	(0.080)	1.116	(0.047)	1.129	(0.038)	*F*(2,92) = 0.02	1.113	0.03
Anterior thalamic radiation MD	1.470	(0.064)	1.471	(0.041)	1.477	(0.039)	*F*(2,92) = 1.48	0.265	0.25
Anterior thalamic radiation AD	2.182	(0.056)	2.181	(0.054)	2.174	(0.062)	*F*(2,92) = 4.27	0.020*^a^	0.43

*Mean and SD of AD, RD, and MD are multiplied ×1000. Significance level p is adjusted for multiple comparison (FDR).*

*Significant at p (adj.) < 0.05.

*^a^The direction of the interaction AD measure; HC > PT-normal > PT-low, respectively (p = 0.047, p < 0.001, p = 0.011).*

**FIGURE 5:**
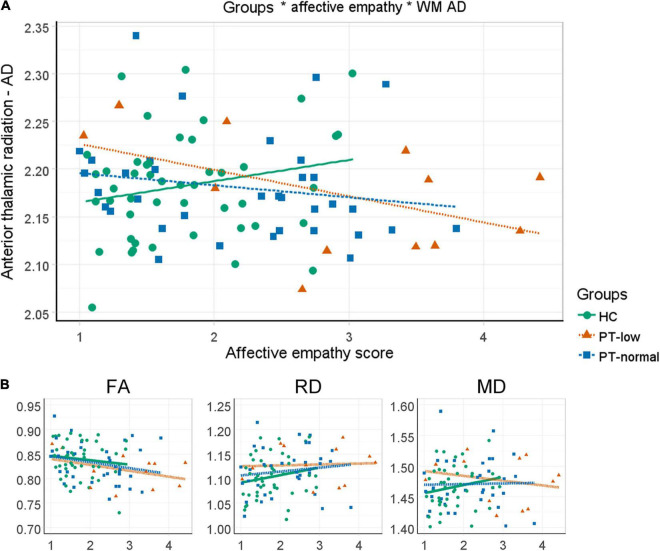
Three-way interactions between emotion recognition, affective empathy and white matter diffusivity measures. **(A)** Anterior thalamic radiation AD*1000 is plotted against affective empathy in healthy controls (green circles/solid line) and patients with normal (blue squares/large dashed line) and low (orange triangles/small dashed line) performance on emotion recognition. Dots are raw values; lines are linear fitted trendlines. **(B)** Similar plots but now for RD*1000, FA and MD*1000 in the anterior thalamic radiation.

In our main analysis, where we investigated the difference between PT-low, PT-normal, and controls on the association between affective empathy and white matter connectivity, we only focused on the diffusion measures in the anterior thalamic radiation. A significant interaction effect was found, indicating that the association between affective empathy and AD differs between PT-low, PT-normal, controls with a moderate effect size [*F*(2,91) = 4.27, *p* = 0.017, *d* = 0.43; Figure 5 and Table 2]. Pairwise analysis revealed that all three groups differed significantly from each other. In controls, higher ratings of affective empathy were related to higher AD while in patients this association was reversed. Moreover, a steeper slope was found in PT-low compared to PT-normal, indicating a stronger relationship between lower level of affective empathy and higher AD in PT-low compared with PT-normal.

IQ did not correlate significantly with level of affective empathy in the total sample (*r* = −0.11; *p* = 0.260), but we did find a significant correlation with emotion recognition performance (*r* = 0.33; *p* = 0.001). *Post hoc* analysis shows that this relation was mainly driven by patients, however, the correlations did not differ significantly between controls (*r* = 0.13; *p* = 0.385) and patients (*r* = 0.34, *p* = 0.02; difference Fishers *r* to *z* = 1.04; *p* = 0.149). Adding IQ as covariate did not essentially change our findings (Supplementary Table 9).

## Discussion

Previous studies have compared patients with schizophrenia and controls on the association between one emotion processing task and white matter structural connectivity (42, 43). Our aim was to go one step further and compare the association between multiple emotion processing tasks and white matter diffusivity between groups. Our main findings are two-fold. First, in contrast to our hypothesis, we showed that patients who have difficulty recognizing emotions from faces experience stronger feelings of affective empathy, reflected in increased, instead of decreased, empathic concern and increased personal distress. Second, in patients with poor emotion recognition capacity, the negative relationship between higher affective empathy and lower AD in the anterior thalamic radiation was stronger than in patients with normal emotion recognition capacity (i.e., who perform in the same range on emotion recognition as controls). In contrast, controls showed a positive association between AD in the anterior thalamic radiation and affective empathy.

To interpret these findings, we must first understand why not only experienced personal distress, but also experiences of empathic concern are stronger in patients than in controls. For this study, we borrowed the subscales items from the IRI (21). Empathic concern represents the tendency to feel warmth, compassion, or concern for the individual in the movie. Personal distress reflects the level of distress one feels while watching the character in the movie in an emotional situation. In line with findings from self-reports [i.e., IRI; (20, 44, 45)], we found increased personal distress in patients. In contrast, we found higher levels of empathic concern, while previous studies showed that self-reported empathic concern usually is lower in patients than in controls (22). A possible explanation for the discrepancy between previous findings and ours might be that we asked participants to rate in-the-moment emotional experiences immediately after watching a movie of an individual in an emotional situation. Our findings may suggest that patients’ judgments on experienced empathic concern is different when taken as *in the moment* assessments of experiences compared to self-reports where one is asked to *reflect* upon a situation and their experiences. Thus, instead of not being able to experience such feelings, as has been suggested from the self-report studies, it may be that patients particularly have problems reflecting on them. Future studies assessing empathic concern and personal distress from both self-report and performance measures in the same individuals are necessary to confirm this hypothesis.

Our finding of poor emotional face recognition in patients is in line with previous literature, particularly with respect to negative expressions (i.e., fear) (46, 47). Additionally, both groups benefited from the contextual information that was provided when viewing the film as emotion recognition performance increased. This corroborates with a study showing that experimentally induced emotional states improved the ability to recognize emotions from faces (48).

Our first main finding suggests that, within patients, a higher level of affective empathy (taking empathic concern and personal distress together) was particularly pronounced in those with deficits in emotional face recognition. It confirms findings of a recent study in healthy individuals, reporting a small but significant negative relationship between emotion perception and affective empathy (23). It is important to note that affective empathy can also be experienced without the conscious identification or recognition of other’s emotions (15, 49, 50), possibly explaining the relatively low association. The question remains what the underlying mechanism is of the association between poor emotional face recognition and increased affective empathy. It may be that having difficulties with recognizing an emotional expression of one’s interaction partner brings feelings of uncertainty to the situation, which may lead to an increase in personal distress. However, this does not explain the higher level of empathic concern we found in patients. Alternatively, it may simply reflect the severity of social cognitive deficits in general, such that low performance on one social cognitive task increases the likelihood of deviating on another cognitive task (51, 52). However, this is not a satisfying explanation either, as it does not explain why affective empathy experiences were more intense in patients and not less intense. Finally, illness severity may play a role, as patients with poor ability to recognize emotional facial expression had significantly more severe symptoms, particularly negative symptoms, than patients whose performance was similar to that of controls. Importantly, general neurocognitive functioning, as indicated by a measure of premorbid IQ, did not explain the association between the two social cognitive tasks.

An association between poor emotional face recognition and increased levels of affective empathy within patients with schizophrenia may imply that both constructs share an underlying neural mechanism. Indeed, our second main finding suggests that in patients with lower emotion recognition performance a negative relationship exists between experienced affective empathy and AD in the anterior thalamic radiation. This association was stronger than in patients whose performance on emotion recognition is in the same range as controls. Moreover, controls showed a positive association between affective empathy and AD in the anterior thalamic radiation. That the anterior thalamic radiation is involved in emotion processing may not be surprising. It consists of fibers connecting the medial dorsal thalamic nuclei with the frontal cortex, and both the frontal cortex and the thalamus have been implicated in emotion processing capacity in schizophrenia patients. For example, increased activation in the thalamus was correlated with increased empathic accuracy in patients (53). Also in schizophrenia patients, a correlation of the anterior thalamic radiation dysconnectivity with lower emotion recognition performance (54, 55) and with a lower capacity to shift oneself into feelings of fictional characters (i.e., the fantasy scale of the IRI) has been reported (43). Because decreased AD has been linked to axonal injury or less coherent orientation of axons (56), these findings suggest that axon disruption of the fronto-thalamic fibers may be the biological mechanism underlying the association between poor emotion recognition and higher affective empathy.

Previous studies on empathy and white matter connectivity in schizophrenia patients also reported other fibers than the anterior thalamic radiation to be involved in an affective empathy neural network, such as the splenium of the corpus callosum and the inferior longitudinal fascicles (connecting the anterior temporal lobe with the occipital lobe) (43, 57). Based on this available evidence and our findings, one could argue that there exists a network of frontal, parietal, and thalamic areas and connecting white matter fibers that are implicated in abnormal emotional processing in schizophrenia. Similarly, deficits in this network have been shown in patients with autism spectrum disorder (58) and mild cognitive impairment or Alzheimer’s disease (59) in relation to poor affective empathy. Of note, these latter studies used self-report measures of affective empathy.

A strength of our study is the reasonably sized sample, which gives sufficient power to detect effects with moderate effect sizes. Moreover, the application of performance-based measures instead of self-reports of affective empathy may add important new leads to understand cognitive and neural processes underlying emotional processing. Some limitations should be taken into consideration when interpreting our findings. First, the findings reported here were stringently corrected for multiple testing, which reduces the risk of type-I error but may introduce the risk of type-II error. Second, the movie clips are more natural than the static illustrations that have been used in other studies, but our films clips were mute. Adding sound would have further improved the ecological validity of the task. However, even with sound, such film clips are still substantially different from real life social experiences. Third, we ran our analyses on the mean diffusivity measures of both hemispheres and did not consider the potential differential role each hemisphere plays in explaining the patients’ problems with emotion recognition and affective empathy. Although altered asymmetry of FA has been found in patients with schizophrenia (60), there is yet little evidence showing that this asymmetry plays a main role in the emotion processing deficits. Finally, future work should quantify the contribution of extracellular free-water to the signal, which could give an indication as to whether the observed effects are caused by microstructural changers or extracellular (potentially neuroinflammatory) mechanisms (61). This may be particularly relevant for the anterior thalamic radiation as it is located in close proximity to the ventricles and is therefore vulnerable to free-water contamination.

## Conclusion

The current study indicates that schizophrenia patients who have deficits in emotion recognition, experience a higher level of -in the moment- affective empathy (not only personal distress, but also empathic concern) than patients with normal emotion recognition and healthy controls. Lower AD, an indication of axonal damage, of the anterior thalamic radiation may underlie this interaction. In patients with poor emotion recognition, the negative association between AD in the anterior thalamic radiation and affective empathy was stronger than in patients with normal emotion recognition capacity. This may imply that axonal damage in fronto-thalamic structural connections, as part of a larger fronto-parietal-thalamic network, explains the association between poor emotion recognition and higher levels of affective empathy in patients with schizophrenia.

## Data Availability Statement

The datasets presented in this article are not readily available because informed consent does not provide an explicit consent to share the data. Requests to access the datasets should be directed to the corresponding author.

## Ethics Statement

The studies involving human participants were reviewed and approved by the Medical Ethics Committee at University Medical Centre Utrecht. The patients/participants provided their written informed consent to participate in this study.

## Author Contributions

NH and MK contributed to the conception and design of the study, and performed the statistical analysis. MP and DH organized the database. MK wrote the first draft of the manuscript. NH, MP, DH, and RK wrote the sections of the manuscript. All authors contributed to manuscript revision, read, and approved the submitted version.

## Conflict of Interest

The authors declare that the research was conducted in the absence of any commercial or financial relationships that could be construed as a potential conflict of interest.

## Publisher’s Note

All claims expressed in this article are solely those of the authors and do not necessarily represent those of their affiliated organizations, or those of the publisher, the editors and the reviewers. Any product that may be evaluated in this article, or claim that may be made by its manufacturer, is not guaranteed or endorsed by the publisher.

## Footnotes

1
http://proefpersonen.nl

2
http://freesurfer.net/

3
http://www.R-project.org
