# Supplementary material for: The Association Between Emotion Recognition, Affective Empathy, and Structural Connectivity in Schizophrenia Patients
Source: Front Psychiatry. 2022 Jun 15;13:910985. doi: 10.3389/fpsyt.2022.910985 (PMC9240782; doi:10.3389/fpsyt.2022.910985)
Supplement: Supplementary file 1 [file Data_Sheet_1.docx]

**Supplementary Material**

**The Association Between Emotion Recognition, Affective Empathy, and Structural Connectivity in Schizophrenia Patients**

Martijn G.J.C. Koevoets ^1^, Merel Prikken ^1^, Doesjka A. Hagenaar^1,2^, René S Kahn^1,3^, Neeltje E.M. van Haren ^1,2^

^1^ Department of Psychiatry, University Medical Center Utrecht Brain Center, Utrecht University, Utrecht, The Netherlands

^2^ Department of Child and Adolescent Psychiatry/Psychology, Erasmus Medical Centre Sophia, Rotterdam, The Netherlands

^3^ Icahn School of Medicine at Mount Sinai, Department of Psychiatry, New York, New York, USA

**FIGURES**

**
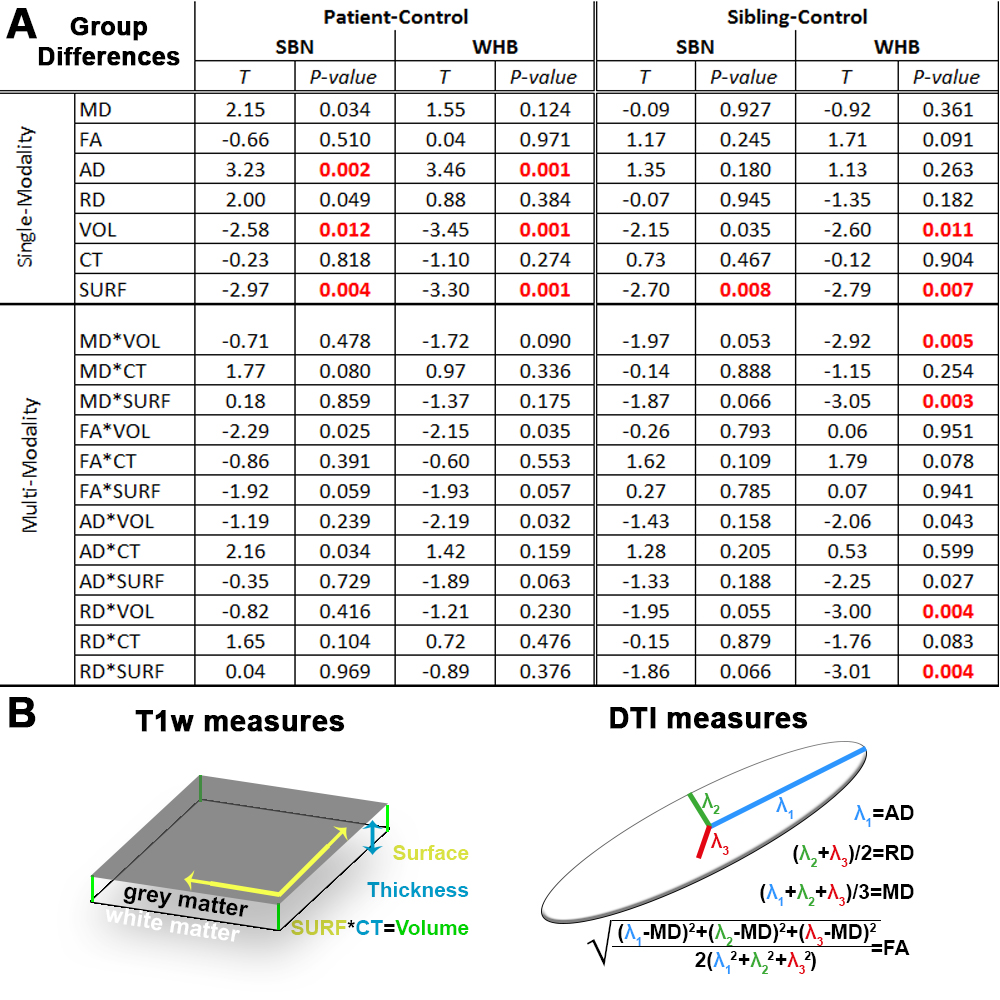
Supplementary Figure 1.** Relationship between Fractional Anisotropy (FA), Axial Diffusivity (AD), Radial Diffusivity (RD), and Mean Diffusivity (MD), represented by a 3D-elipsoid.

Eigenvalues and formulas are shown in corresponding colours

**Supplementary Figure 2.** Correlation between personal distress and empathic concern in patients and controls.

**
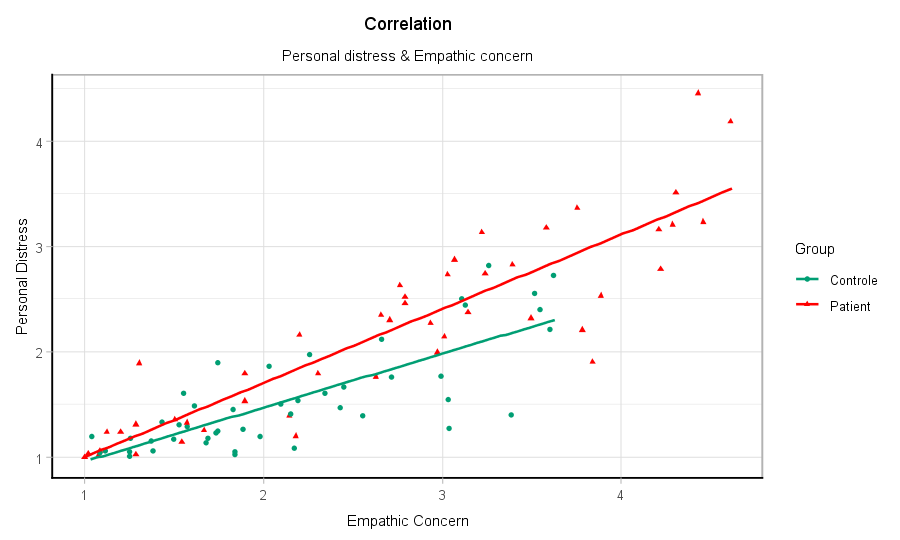
**

*Empathic concern and personal distress were highly correlated in both groups (patients: Pearson r=0.78, p<0.001; controls: r=0.89, p<0.001*

**TABLES**

**Supplementary Table 1.** Demographic and clinical information of included and excluded participants

|  | **Included participants (n=94)** | | **Excluded participants^1^ (n=43)** | | **Controls (Incl vs Excl)** | | **Patients (Incl vs Excl)** | |
| --- | --- | --- | --- | --- | --- | --- | --- | --- |
|  | **Controls (n=47)** | **Patients (n=47)** | **Controls (n=22)** | **Patients (n=20)** | **Test statistic** | **p** | **Test statistic** | **p** |
|  | Mean (sd) or N (%) | Mean (sd) or N (%) | Mean (sd) or N (%) | Mean (sd) or N (%) | **(chi/t)** |  | **(chi/t)** |  |
| Age [years] | 32.88 (7.91) | 35.88 (8.24) | 32.95 (7.33) | 36.88 (7.86) | t=0.04 | 0.972 | t=0.476 | 0.635 |
| Range | 19.08 - 48.75 | 22.17 - 50.92 | 19.17 - 45.33 | 25.33 - 49.42 |  |  |  |  |
| Sex [N: M/F and F%] | 43/4 - 8.5% | 40/7 - 14.9% | 20/2 10% | 17/3 15% | χ2 (2) =0.01 | 0.936 | χ2 (2) <0.01 | 0.991 |
| Subject education [years] | 15.8 (1.9) | 16.1 (1.5) | 15.9 (2.5) | 14.8(2.3) | t=0.18 | 0.855 | t=1.72 | 0.091 |
| Parental education [years] | 13.6 (3.3) | 14.2 (4.4) | 14 (2.7) | 14.2 (2.2) | t=0.50 | 0.622 | t=0.04 | 0.968 |
| Premorbid IQ | 103.65 (7.46) | 98.74 (8.84) | 100.77 (6.52) | 98.95(8.83) | t=1.55 | 0.125 | t=0.94 | 0.352 |
| MR Scanner (N: A/B/not scanned and B%) | 31/16/0 - 34.0% | 35/12/0 - 32.4% | 10/5/6 - 23.8% | 11/6/3 - 42.9% | χ2 (2) <0.01 | 0.960 | χ2 (2) =0.59 | 0.443 |
| PANSS total |  | 52.13 (11.06) |  | 50.33 (5.31) | t=0.65 | 0.517 | t=0.65 | 0.517 |
| Positive |  | 13.28 (3.90) |  | 13.39 (3.86) | t=-0.07 | 0.943 | t=-0.07 | 0.943 |
| Negative |  | 13.49 (4.99) |  | 12.33 (3.55) | t=0.92 | 0.363 | t=0.92 | 0.363 |
| General |  | 25.36 (5.59) |  | 24.61 (5.60) | t=0.50 | 0.622 | t=0.50 | 0.622 |
| Illness duration [years] |  | 14.10 (7.98) |  | 13.73 (8.22) | t=0.25 | 0.803 | t=0.25 | 0.803 |
| Medication Type (N) |  |  |  |  | χ2 (4) =0.41 | 0.613 | χ2 (4) =0.41 | 0.613 |
| Typical |  | 2 |  | 2 |  |  |  |  |
| Atypical |  | 40 |  | 19 |  |  |  |  |
| Both |  | 1 |  | 2 |  |  |  |  |
| No medication |  | 4 |  | 0 |  |  |  |  |
|  |  |  |  |  |  |  |  |  |
| *^1^Reasons for exclusion: excessive MRI movement (n=9), incomplete or missing questionnaires/tasks (n=31), family history of psychosis (n=2).* | | | | | |  |  |  |

**Supplementary Table 2.** Differences in DTI measures between the two scanners (A v. B).

|  | *Mean ± sd* | | | | | | *Statistics* | | | |  |
| --- | --- | --- | --- | --- | --- | --- | --- | --- | --- | --- | --- |
|  | **MRI scanner A**  **(n=28)** | | | **MRI scanner B**  **(n=66)** | | | **F** | | **p** | **p (Adjusted)** | **Cohen’s d** |
| *Corpus callosum forceps major* |  | | |  |  |  |  |  |  |  |  |
| AD | 1.451 | ± | 0.053 | 1.448 | ± | 0.044 | F(1,92)= | 0.98 | 0.324 | 0.995 | 0.23 |
| RD | 0.447 | ± | 0.037 | 0.462 | ± | 0.098 | F(1,92)= | 0.08 | 0.784 | 0.995 | 0.06 |
| MD | 0.781 | ± | 0.027 | 0.791 | ± | 0.066 | F(1,92)< | 0.01 | 0.995 | 0.995 | 0.001 |
| FA | 0.628 | ± | 0.035 | 0.617 | ± | 0.071 | F(1,92)= | 0.27 | 0.603 | 0.995 | 0.12 |
| *Corpus callosum forceps minor* |  |  |  |  |  |  |  |  |  |  |  |
| AD | 1.329 | ± | 0.038 | 1.335 | ± | 0.052 | F(1,92)= | 0.16 | 0.695 | 0.995 | 0.09 |
| RD | 0.502 | ± | 0.030 | 0.524 | ± | 0.058 | F(1,92)= | 0.01 | 0.929 | 0.995 | 0.02 |
| MD | 0.778 | ± | 0.024 | 0.794 | ± | 0.037 | F(1,92)= | 0.01 | 0.923 | 0.995 | 0.02 |
| FA | 0.544 | ± | 0.027 | 0.526 | ± | 0.056 | F(1,92)= | 0.02 | 0.877 | 0.995 | 0.04 |
| *Ant. thalamic radiat.* |  |  |  |  |  |  |  |  |  |  |  |
| AD | 2.195 | ± | 0.047 | 2.175 | ± | 0.058 | F(1,92)= | 0.28 | 0.600 | 0.995 | 0.12 |
| RD | 1.112 | ± | 0.042 | 1.118 | ± | 0.073 | F(1,92)= | 0.05 | 0.826 | 0.995 | 0.05 |
| MD | 1.473 | ± | 0.038 | 1.470 | ± | 0.059 | F(1,92)= | 0.13 | 0.721 | 0.995 | 0.08 |
| FA | 0.839 | ± | 0.033 | 0.827 | ± | 0.052 | F(1,92)= | 0.03 | 0.867 | 0.995 | 0.04 |
| *Cingulum angular gyrus* |  |  |  |  |  |  |  |  |  |  |  |
| AD | 2.316 | ± | 0.106 | 2.280 | ± | 0.116 | F(1,92)= | 0.88 | 0.350 | 0.995 | 0.21 |
| RD | 1.280 | ± | 0.093 | 1.295 | ± | 0.099 | F(1,92)= | 0.36 | 0.552 | 0.995 | 0.14 |
| MD | 1.625 | ± | 0.083 | 1.624 | ± | 0.090 | F(1,92)< | 0.01 | 0.967 | 0.995 | 0.01 |
| FA | 0.747 | ± | 0.073 | 0.725 | ± | 0.073 | F(1,92)= | 1.38 | 0.243 | 0.995 | 0.27 |
| *Ccingulum cingulate gyrus* |  |  |  |  |  |  |  |  |  |  |  |
| AD | 2.457 | ± | 0.120 | 2.437 | ± | 0.150 | F(1,92)= | 2.60 | 0.110 | 0.995 | 0.37 |
| RD | 0.966 | ± | 0.092 | 0.956 | ± | 0.161 | F(1,92)= | 0.05 | 0.825 | 0.995 | 0.05 |
| MD | 1.463 | ± | 0.083 | 1.450 | ± | 0.136 | F(1,92)= | 0.19 | 0.663 | 0.995 | 0.10 |
| FA | 1.087 | ± | 0.083 | 1.093 | ± | 0.115 | F(1,92)= | 0.49 | 0.487 | 0.995 | 0.16 |
| *Corticospinal tract* |  |  |  |  |  |  |  |  |  |  |  |
| AD | 2.351 | ± | 0.055 | 2.326 | ± | 0.064 | F(1,92)= | 1.96 | 0.165 | 0.995 | 0.32 |
| RD | 0.946 | ± | 0.049 | 0.945 | ± | 0.115 | F(1,92)= | 0.04 | 0.847 | 0.995 | 0.04 |
| MD | 1.414 | ± | 0.040 | 1.405 | ± | 0.089 | F(1,92)= | 0.27 | 0.602 | 0.995 | 0.12 |
| FA | 1.060 | ± | 0.044 | 1.060 | ± | 0.076 | F(1,92)= | 0.19 | 0.662 | 0.995 | 0.10 |
| *Inf. long. fascicle* |  |  |  |  |  |  |  |  |  |  |  |
| AD | 2.577 | ± | 0.071 | 2.441 | ± | 0.092 | F(1,92)= | 0.83 | 0.364 | 0.995 | 0.21 |
| RD | 1.101 | ± | 0.048 | 1.077 | ± | 0.070 | F(1,92)< | 0.01 | 0.976 | 0.995 | 0.01 |
| MD | 1.593 | ± | 0.044 | 1.532 | ± | 0.053 | F(1,92)= | 0.29 | 0.594 | 0.995 | 0.12 |
| FA | 1.008 | ± | 0.040 | 0.985 | ± | 0.070 | F(1,92)= | 0.16 | 0.688 | 0.995 | 0.09 |
| *Sup. long. fascicle parietal* |  |  |  |  |  |  |  |  |  |  |  |
| AD | 2.186 | ± | 0.067 | 2.130 | ± | 0.063 | F(1,92)= | 0.07 | 0.786 | 0.995 | 0.06 |
| RD | 1.055 | ± | 0.042 | 1.033 | ± | 0.066 | F(1,92)= | 1.80 | 0.183 | 0.995 | 0.31 |
| MD | 1.432 | ± | 0.042 | 1.398 | ± | 0.051 | F(1,92)= | 0.99 | 0.324 | 0.995 | 0.23 |
| FA | 0.910 | ± | 0.039 | 0.908 | ± | 0.065 | F(1,92)= | 1.66 | 0.200 | 0.995 | 0.29 |
| *Sup. long. fascicle temporal* |  |  |  |  |  |  |  |  |  |  |  |
| AD | 2.260 | ± | 0.058 | 2.179 | ± | 0.055 | F(1,92)= | 0.13 | 0.717 | 0.995 | 0.08 |
| RD | 1.043 | ± | 0.045 | 1.022 | ± | 0.071 | F(1,92)= | 0.61 | 0.436 | 0.995 | 0.18 |
| MD | 1.449 | ± | 0.042 | 1.408 | ± | 0.055 | F(1,92)= | 0.28 | 0.599 | 0.995 | 0.12 |
| FA | 0.942 | ± | 0.037 | 0.932 | ± | 0.060 | F(1,92)= | 1.23 | 0.271 | 0.995 | 0.25 |
| *Uncinate fascicle* |  |  |  |  |  |  |  |  |  |  |  |
| AD | 2.325 | ± | 0.060 | 2.299 | ± | 0.065 | F(1,92)= | 0.65 | 0.423 | 0.995 | 0.18 |
| RD | 1.187 | ± | 0.056 | 1.238 | ± | 0.079 | F(1,92)= | 0.41 | 0.522 | 0.995 | 0.15 |
| MD | 1.566 | ± | 0.046 | 1.592 | ± | 0.061 | F(1,92)= | 0.74 | 0.393 | 0.995 | 0.20 |
| FA | 0.830 | ± | 0.050 | 0.777 | ± | 0.060 | F(1,92)= | 0.10 | 0.758 | 0.995 | 0.07 |

FA=Fractional Anisotropy. MD=Mean Diffusivity. AD=Axial Diffusivity. RD=Radial Diffusivity. MD, AD and RD measures were multiplied by 1000. Analyses were corrected for age and sex.

**Supplementary Table 3.** Emotion recognition scores from the picture and after the movie in controls and patients

|  | **Controls** | **Patients** | **Test statistics** | **p** | **Effect size Phi** |  |  |  |
| --- | --- | --- | --- | --- | --- | --- | --- | --- |
| *Emotional face recognition* | *From picture % correct (sd)* | | χ2 (4) |  |  |  |  |  |
| Happy | 100 (0.0) | 95.7 (14.2) | 8.74 | 0.033* | 0.61 |  |  |  |
| Neutral | 91.5 (10.3) | 85.8 (20.3) | 8.29 | 0.141 | 0.59 |  |  |  |
| Sad | 56.2 (10.0) | 53.6 (12.6) | 3.54 | 0.316 | 0.39 |  |  |  |
| Fear | 96.2 (9.3) | 89.7 (11.3) | 12.8 | 0.002* | 0.74 |  |  |  |
| Total | 83.6 (5.0) | 79.0 (8.6) | 14.96 | 0.037* | 0.80 |  |  |  |
|  | *After movie % correct (sd)* | |  |  |  |  |  |  |
| Happy | 99.6 (2.4) | 95.3 (14.2) | 5.22 | 0.157 | 0.47 |  |  |  |
| Neutral | 95.7 (9.3) | 90.0 (19.7) | 5.70 | 0.222 | 0.49 |  |  |  |
| Sad | 93.2 (13.4) | 88.1 (17.5) | 3.73 | 0.444 | 0.40 |  |  |  |
| Fear | 99.1 (6.3) | 97.6 (10.9) | 5.10 | 0.165 | 0.47 |  |  |  |
| Total | 93.2 (5.9) | 89.0 (12.8) | 12.32 | 0.138 | 0.72 |  |  |  |
|  |  |  | **Within groups** | | | | **Between groups** | |
|  | **Controls** | **Patients** | **Controls** | | **Patients** | |  |  |
|  | *% Change (movie-picture)* | | *t* | *p* | *t* | *p* | *t* | *p* |
| Happy | -0.4 | -0.4 | -1.00 | 0.323 | -0.14 | 0.892 | 0.00 | 1.000 |
| Neutral | 4.0 | 4.2 | 6.21 | <0.001* | 2.83 | 0.006* | -0.18 | 0.858 |
| Sad | 37.0 | 34.5 | 15.26 | <0.001* | 10.95 | <0.001* | 0.77 | 0.443 |
| Fear | 2.9 | 7.9 | 11.29 | <0.001* | 9.74 | <0.001* | -1.56 | 0.123 |
| Total | 9.6 | 10.0 | 8.56 | <0.001* | 4.46 | <0.001* | -0.25 | 0.800 |

**Supplementary Table 4.** Difference in diffusivity measures in 10 major white matter tracts between controls and patients.

|  | *Means ± sd* | | | | | | *Statistics* | | | |  |
| --- | --- | --- | --- | --- | --- | --- | --- | --- | --- | --- | --- |
|  | **Controls (n=47)** | | | **Patients**  **(n=47)** | | | **F** | | **p** | **p (Adjusted)** | **Cohens’ d** |
| *Corpus callosum forceps major* |  | | |  |  |  |  |  |  |  |  |
| AD | 1.441 | ± | 0.048 | 1.457 | ± | 0.045 | F(1.92)= | 2.924 | 0.091 | 0.482 | 0.357 |
| RD | 0.454 | ± | 0.105 | 0.461 | ± | 0.058 | F(1.92)= | 0.129 | 0.720 | 0.918 | 0.075 |
| MD | 0.783 | ± | 0.071 | 0.793 | ± | 0.039 | F(1.92)= | 0.670 | 0.415 | 0.853 | 0.171 |
| FA | 0.621 | ± | 0.074 | 0.620 | ± | 0.048 | F(1.92)= | 0.008 | 0.927 | 0.941 | 0.019 |
| *Corpus callosum forceps minor* |  |  |  |  |  |  |  |  |  |  |  |
| AD | 1.324 | ± | 0.047 | 1.342 | ± | 0.048 | F(1.92)= | 3.135 | 0.080 | 0.482 | 0.369 |
| RD | 0.511 | ± | 0.062 | 0.524 | ± | 0.041 | F(1.92)= | 1.419 | 0.237 | 0.822 | 0.248 |
| MD | 0.782 | ± | 0.036 | 0.797 | ± | 0.031 | F(1.92)= | 4.340 | 0.040 | 0.482 | 0.434 |
| FA | 0.534 | ± | 0.059 | 0.528 | ± | 0.038 | F(1.92)= | 0.337 | 0.563 | 0.853 | 0.121 |
| *Ant. thalamic radiat.* |  |  |  |  |  |  |  |  |  |  |  |
| AD | 2.182 | ± | 0.056 | 2.179 | ± | 0.056 | F(1.92)= | 0.080 | 0.778 | 0.918 | 0.059 |
| RD | 1.113 | ± | 0.080 | 1.119 | ± | 0.045 | F(1.92)= | 0.182 | 0.671 | 0.912 | 0.089 |
| MD | 1.470 | ± | 0.064 | 1.472 | ± | 0.040 | F(1.92)= | 0.063 | 0.803 | 0.918 | 0.052 |
| FA | 0.834 | ± | 0.055 | 0.828 | ± | 0.040 | F(1.92)= | 0.346 | 0.558 | 0.853 | 0.123 |
| *Cingulum angular gyrus* |  |  |  |  |  |  |  |  |  |  |  |
| AD | 2.278 | ± | 0.118 | 2.305 | ± | 0.108 | F(1.92)= | 1.330 | 0.252 | 0.822 | 0.240 |
| RD | 1.283 | ± | 0.110 | 1.298 | ± | 0.082 | F(1.92)= | 0.529 | 0.469 | 0.853 | 0.152 |
| MD | 1.615 | ± | 0.095 | 1.634 | ± | 0.079 | F(1.92)= | 1.058 | 0.307 | 0.822 | 0.214 |
| FA | 0.732 | ± | 0.079 | 0.731 | ± | 0.067 | F(1.92)= | 0.006 | 0.941 | 0.941 | 0.016 |
| *Cingulum cingulate gyrus* |  |  |  |  |  |  |  |  |  |  |  |
| AD | 2.453 | ± | 0.159 | 2.433 | ± | 0.121 | F(1.92)= | 0.487 | 0.487 | 0.853 | 0.145 |
| RD | 0.943 | ± | 0.186 | 0.975 | ± | 0.083 | F(1.92)= | 1.123 | 0.292 | 0.822 | 0.221 |
| MD | 1.447 | ± | 0.161 | 1.461 | ± | 0.065 | F(1.92)= | 0.316 | 0.576 | 0.853 | 0.117 |
| FA | 1.112 | ± | 0.119 | 1.070 | ± | 0.088 | F(1.92)= | 3.733 | 0.057 | 0.482 | 0.403 |
| *Corticospinal tract* |  |  |  |  |  |  |  |  |  |  |  |
| AD | 2.328 | ± | 0.066 | 2.339 | ± | 0.059 | F(1.92)= | 0.667 | 0.416 | 0.853 | 0.170 |
| RD | 0.950 | ± | 0.130 | 0.941 | ± | 0.055 | F(1.92)= | 0.167 | 0.684 | 0.912 | 0.085 |
| MD | 1.409 | ± | 0.102 | 1.407 | ± | 0.042 | F(1.92)= | 0.019 | 0.890 | 0.941 | 0.029 |
| FA | 1.056 | ± | 0.081 | 1.064 | ± | 0.052 | F(1.92)= | 0.353 | 0.554 | 0.853 | 0.124 |
| *Inf. long. fascicle* |  |  |  |  |  |  |  |  |  |  |  |
| AD | 2.475 | ± | 0.116 | 2.488 | ± | 0.096 | F(1.92)= | 0.472 | 0.494 | 0.853 | 0.143 |
| RD | 1.081 | ± | 0.065 | 1.087 | ± | 0.065 | F(1.92)= | 0.200 | 0.656 | 0.912 | 0.093 |
| MD | 1.546 | ± | 0.058 | 1.554 | ± | 0.058 | F(1.92)= | 0.579 | 0.449 | 0.853 | 0.159 |
| FA | 0.993 | ± | 0.066 | 0.991 | ± | 0.062 | F(1.92)= | 0.030 | 0.862 | 0.941 | 0.036 |
| *Sup. long. fascicle parietal* |  |  |  |  |  |  |  |  |  |  |  |
| AD | 2.136 | ± | 0.076 | 2.158 | ± | 0.061 | F(1.92)= | 2.916 | 0.091 | 0.482 | 0.356 |
| RD | 1.033 | ± | 0.053 | 1.046 | ± | 0.068 | F(1.92)= | 1.051 | 0.308 | 0.822 | 0.214 |
| MD | 1.400 | ± | 0.050 | 1.416 | ± | 0.051 | F(1.92)= | 2.567 | 0.113 | 0.501 | 0.334 |
| FA | 0.911 | ± | 0.049 | 0.907 | ± | 0.066 | F(1.92)= | 0.113 | 0.738 | 0.918 | 0.070 |
| *Sup. long. fascicle temporal* |  |  |  |  |  |  |  |  |  |  |  |
| AD | 2.194 | ± | 0.072 | 2.213 | ± | 0.061 | F(1.92)= | 2.824 | 0.096 | 0.482 | 0.350 |
| RD | 1.027 | ± | 0.070 | 1.030 | ± | 0.060 | F(1.92)= | 0.069 | 0.794 | 0.918 | 0.055 |
| MD | 1.416 | ± | 0.060 | 1.424 | ± | 0.048 | F(1.92)= | 0.674 | 0.414 | 0.853 | 0.171 |
| FA | 0.934 | ± | 0.052 | 0.936 | ± | 0.057 | F(1.92)= | 0.012 | 0.913 | 0.941 | 0.023 |
| *Uncinate fascicle* |  |  |  |  |  |  |  |  |  |  |  |
| AD | 2.293 | ± | 0.066 | 2.320 | ± | 0.060 | F(1.92)= | 4.421 | 0.038 | 0.482 | 0.438 |
| RD | 1.212 | ± | 0.079 | 1.234 | ± | 0.073 | F(1.92)= | 2.133 | 0.148 | 0.591 | 0.305 |
| MD | 1.572 | ± | 0.058 | 1.596 | ± | 0.056 | F(1.92)= | 4.188 | 0.044 | 0.482 | 0.427 |
| FA | 0.797 | ± | 0.063 | 0.788 | ± | 0.061 | F(1.92)= | 0.588 | 0.445 | 0.853 | 0.160 |

FA=Fractional Anisotropy. MD=Mean Diffusivity. AD=Axial Diffusivity. RD=Radial Diffusivity. MD, AD and RD measures were multiplied by 1000. Analyses were corrected for age, sex, and scanner.

**Supplementary Table 5.** Demographic and clinical information of PT-low and PT-normal

|  | **PT-low (n=12)** | **PT-normal (n=35)** | **Test statistics** | **p** |
| --- | --- | --- | --- | --- |
|  | Mean (sd) or N (%) | Mean (sd) or N (%) |  |  |
|  |  |  |  |  |
| Age (years) | 31.67 (10.86) | 38.71 (16.04) | t=-0.95 | 0.354 |
| Sex (M/F) | 12/0 | 28/7 | χ2 (2) =2.76 | 0.097 |
| Subject education (years) | 15.18 (1.40) | 16.71 (2.09) | t=-2.78 | 0.010* |
| Parental education (years) | 12.85 (5.24) | 14.68 (4.14) | t=-1.01 | 0.333 |
| Premorbid IQ | 94.45 (9.04) | 100.09 (8.47) | t=-1.83 | 0.086 |
| PANSS Total | 58.92 (10.64) | 49.80 (10.34) | t=2.58 | 0.019* |
| Positive | 14.42 (4.96) | 12.89 (3.47) | t=0.99 | 0.339 |
| Negative | 16.92 (4.34) | 12.31 (4.70) | t=3.10 | 0.005* |
| General | 27.58 (5.71) | 24.60 (5.42) | t=1.58 | 0.131 |
| Illness duration (years) | 10.27 (7.57) | 15.34 (8.53) | t=-1.77 | 0.095 |
| Medication Type |  |  | χ2 (5) =4.12 | 0.042* |
| Atypical | 8 (66.7) | 32 (91.4) |  |  |
| Both | 0 | 1 (2.9) |  |  |
| No medication | 3 (25.0) | 0 |  |  |
| Other | 0 | 1 (2.9) |  |  |
| Typical | 1 (8.3) | 1 (2.9) |  |  |

**Supplementary Table 6.** Differences in affective empathy subscales scores between patients with low scores on emotion recognition (PT-low), patients with comparable scores as controls (PT-normal), and controls.

|  | *Mean (SD)* | | | | | | | | | *Statistics* | | |  | | | |
| --- | --- | --- | --- | --- | --- | --- | --- | --- | --- | --- | --- | --- | --- | --- | --- | --- |
|  |  |  |  |  |  |  |  |  |  |  |  |  | *HC v PT-normal* | *PT-normal v PT-low* | | *HC PT-low* |
|  | **HC** | | | **PT-normal** | | | **PT-low** | | | **F** | **p** | **Pairwise** | **p** | **p** | | **p** |
| *Affective Empathy* | 1.78 | ± | 0.55 | 2.17 | ± | 0.78 | 2.89 | ± | 1.11 | 11.97 | <0.001 | HC <PT-norm <PT-low | 0.044 | 0.010 | | <0.001 |
| *Personal distress* | 1.55 | ± | 0.50 | 1.98 | ± | 0.69 | 2.77 | ± | 1.10 | 16.84 | <0.001 | HC <PT-norm <PT-low | 0.011 | 0.002 | | <0.001 |
| neutral | 1.13 | ± | 0.21 | 1.28 | ± | 0.44 | 2.10 | ± | 0.74 | 27.88 | <0.001 | HC&PT-norm <PT-low | 0.242 | <0.001 | | <0.001 |
| happy | 1.09 | ± | 0.23 | 1.31 | ± | 0.46 | 1.92 | ± | 0.76 | 18.54 | <0.001 | HC&PT-norm <PT-low | 0.063 | <0.001 | | <0.001 |
| sad | 1.79 | ± | 0.79 | 2.42 | ± | 1.12 | 3.41 | ± | 1.57 | 12.47 | <0.001 | HC <PT-norm <PT-low | 0.021 | 0.015 | | <0.001 |
| fear | 2.07 | ± | 1.02 | 2.90 | ± | 1.31 | 3.59 | ± | 1.68 | 9.29 | <0.001 | HC <PT-norm <PT-low | 0.009 | 0.217 | | 0.001 |
| *Empathic concern* | 2.15 | ± | 0.76 | 2.51 | ± | 1.02 | 3.22 | ± | 1.23 | 6.64 | 0.002 | HC <PT-norm <PT-low | 0.050 | 0.049 | | 0.002 |
| neutral | 1.45 | ± | 0.50 | 1.69 | ± | 0.86 | 2.77 | ± | 1.24 | 14.16 | <0.001 | HC&PT-norm <PT-low | 0.341 | 0.000 | | <0.001 |
| happy | 3.17 | ± | 1.27 | 3.53 | ± | 1.50 | 3.65 | ± | 1.46 | 0.95 | 0.390 | - | - | | - | - |
| sad | 2.21 | ± | 0.94 | 2.62 | ± | 1.19 | 3.33 | ± | 1.25 | 5.46 | 0.006 | HC<PT-low | 0.217 | | 0.122 | 0.005 |
| fear | 1.60 | ± | 0.53 | 1.91 | ± | 0.70 | 2.74 | ± | 1.08 | 13.44 | <0.001 | HC&PT-norm <PT-low | 0.112 | | 0.001 | <0.001 |

**Supplementary Table 7.** Difference in all diffusivity measures between controls and patients who score in the same range as controls (PT-normal) and those who score lower than controls (PT-low) on emotion recognition in 10 major white matter tracts.

|  | *Means* ±*sd* | | | | | | | | | *Statistics* | | | |
| --- | --- | --- | --- | --- | --- | --- | --- | --- | --- | --- | --- | --- | --- |
|  | **Controls (n=47)** | | | **PT-normal (n=35)** | | | **PT-low**  **(n=12)** | | | **F** | | **p** | **p (Adjusted)** |
| *Corpus callosum forceps major* |  |  |  |  |  |  |  |  |  |  |  |  |  |
| AD | 1.441 | ± | 0.048 | 1.452 | ± | 0.056 | 1.459 | ± | 0.041 | F(2,92)= | 1.561 | 0.216 | 0.588 |
| RD | 0.454 | ± | 0.105 | 0.455 | ± | 0.043 | 0.463 | ± | 0.063 | F(2,92)= | 0.105 | 0.900 | 0.934 |
| MD | 0.783 | ± | 0.071 | 0.787 | ± | 0.024 | 0.795 | ± | 0.043 | F(2,92)= | 0.417 | 0.660 | 0.930 |
| FA | 0.621 | ± | 0.074 | 0.623 | ± | 0.044 | 0.619 | ± | 0.050 | F(2,92)= | 0.022 | 0.979 | 0.979 |
| *Corpus callosum forceps minor* |  |  |  |  |  |  |  |  |  |  |  |  |  |
| AD | 1.324 | ± | 0.047 | 1.342 | ± | 0.039 | 1.342 | ± | 0.051 | F(2,92)= | 1.551 | 0.218 | 0.588 |
| RD | 0.511 | ± | 0.062 | 0.522 | ± | 0.040 | 0.525 | ± | 0.042 | F(2,92)= | 0.714 | 0.493 | 0.842 |
| MD | 0.782 | ± | 0.036 | 0.795 | ± | 0.033 | 0.797 | ± | 0.031 | F(2,92)= | 2.157 | 0.122 | 0.588 |
| FA | 0.534 | ± | 0.059 | 0.531 | ± | 0.028 | 0.527 | ± | 0.041 | F(2,92)= | 0.192 | 0.825 | 0.930 |
| *Ant. thalamic radiat.* |  |  |  |  |  |  |  |  |  |  |  |  |  |
| AD | 2.182 | ± | 0.056 | 2.174 | ± | 0.062 | 2.181 | ± | 0.054 | F(2,92)= | 0.099 | 0.906 | 0.934 |
| RD | 1.113 | ± | 0.080 | 1.129 | ± | 0.038 | 1.116 | ± | 0.047 | F(2,92)= | 0.259 | 0.772 | 0.930 |
| MD | 1.470 | ± | 0.064 | 1.477 | ± | 0.039 | 1.471 | ± | 0.041 | F(2,92)= | 0.093 | 0.911 | 0.934 |
| FA | 0.834 | ± | 0.055 | 0.817 | ± | 0.035 | 0.831 | ± | 0.041 | F(2,92)= | 0.558 | 0.574 | 0.909 |
| *Cingulum angular gyrus* |  |  |  |  |  |  |  |  |  |  |  |  |  |
| AD | 2.278 | ± | 0.118 | 2.286 | ± | 0.150 | 2.311 | ± | 0.092 | F(2,92)= | 0.872 | 0.422 | 0.842 |
| RD | 1.283 | ± | 0.110 | 1.311 | ± | 0.083 | 1.293 | ± | 0.083 | F(2,92)= | 0.404 | 0.669 | 0.930 |
| MD | 1.615 | ± | 0.095 | 1.636 | ± | 0.090 | 1.633 | ± | 0.076 | F(2,92)= | 0.530 | 0.591 | 0.909 |
| FA | 0.732 | ± | 0.079 | 0.709 | ± | 0.076 | 0.738 | ± | 0.064 | F(2,92)= | 0.722 | 0.489 | 0.842 |
| *Cingulum cingulate gyrus* |  |  |  |  |  |  |  |  |  |  |  |  |  |
| AD | 2.453 | ± | 0.159 | 2.430 | ± | 0.111 | 2.433 | ± | 0.126 | F(2,92)= | 0.243 | 0.784 | 0.930 |
| RD | 0.943 | ± | 0.186 | 0.994 | ± | 0.084 | 0.969 | ± | 0.083 | F(2,92)= | 0.688 | 0.505 | 0.842 |
| MD | 1.447 | ± | 0.161 | 1.473 | ± | 0.071 | 1.457 | ± | 0.064 | F(2,92)= | 0.226 | 0.798 | 0.930 |
| FA | 1.112 | ± | 0.119 | 1.051 | ± | 0.079 | 1.077 | ± | 0.091 | F(2,92)= | 2.111 | 0.127 | 0.588 |
| *Corticospinal tract* |  |  |  |  |  |  |  |  |  |  |  |  |  |
| AD | 2.328 | ± | 0.066 | 2.336 | ± | 0.053 | 2.339 | ± | 0.061 | F(2,92)= | 0.342 | 0.711 | 0.930 |
| RD | 0.950 | ± | 0.130 | 0.959 | ± | 0.051 | 0.935 | ± | 0.056 | F(2,92)= | 0.346 | 0.709 | 0.930 |
| MD | 1.409 | ± | 0.102 | 1.418 | ± | 0.028 | 1.403 | ± | 0.046 | F(2,92)= | 0.178 | 0.837 | 0.930 |
| FA | 1.056 | ± | 0.081 | 1.044 | ± | 0.058 | 1.071 | ± | 0.048 | F(2,92)= | 0.895 | 0.412 | 0.842 |
| *Inf. long. fascicle* |  |  |  |  |  |  |  |  |  |  |  |  |  |
| AD | 2.475 | ± | 0.116 | 2.462 | ± | 0.102 | 2.496 | ± | 0.094 | F(2,92)= | 0.948 | 0.391 | 0.842 |
| RD | 1.081 | ± | 0.065 | 1.117 | ± | 0.074 | 1.077 | ± | 0.060 | F(2,92)= | 1.915 | 0.153 | 0.588 |
| MD | 1.546 | ± | 0.058 | 1.566 | ± | 0.067 | 1.550 | ± | 0.055 | F(2,92)= | 0.703 | 0.498 | 0.842 |
| FA | 0.993 | ± | 0.066 | 0.955 | ± | 0.065 | 1.003 | ± | 0.057 | F(2,92)= | 2.737 | 0.070 | 0.588 |
| *Sup. long. fascicle parietal* |  |  |  |  |  |  |  |  |  |  |  |  |  |
| AD | 2.136 | ± | 0.076 | 2.137 | ± | 0.077 | 2.165 | ± | 0.054 | F(2,92)= | 2.309 | 0.105 | 0.588 |
| RD | 1.033 | ± | 0.053 | 1.069 | ± | 0.059 | 1.038 | ± | 0.070 | F(2,92)= | 1.754 | 0.179 | 0.588 |
| MD | 1.400 | ± | 0.050 | 1.425 | ± | 0.052 | 1.413 | ± | 0.051 | F(2,92)= | 1.538 | 0.221 | 0.588 |
| FA | 0.911 | ± | 0.049 | 0.877 | ± | 0.056 | 0.917 | ± | 0.067 | F(2,92)= | 2.163 | 0.121 | 0.588 |
| *Sup. long. fascicle temporal* |  |  |  |  |  |  |  |  |  |  |  |  |  |
| AD | 2.194 | ± | 0.072 | 2.204 | ± | 0.072 | 2.216 | ± | 0.058 | F(2,92)= | 1.590 | 0.210 | 0.588 |
| RD | 1.027 | ± | 0.070 | 1.059 | ± | 0.058 | 1.020 | ± | 0.058 | F(2,92)= | 1.689 | 0.191 | 0.588 |
| MD | 1.416 | ± | 0.060 | 1.441 | ± | 0.047 | 1.419 | ± | 0.048 | F(2,92)= | 1.182 | 0.311 | 0.779 |
| FA | 0.934 | ± | 0.052 | 0.907 | ± | 0.056 | 0.945 | ± | 0.054 | F(2,92)= | 2.288 | 0.108 | 0.588 |
| *Uncinate fascicle* |  |  |  |  |  |  |  |  |  |  |  |  |  |
| AD | 2.293 | ± | 0.066 | 2.314 | ± | 0.074 | 2.322 | ± | 0.056 | F(2,92)= | 2.269 | 0.109 | 0.588 |
| RD | 1.212 | ± | 0.079 | 1.228 | ± | 0.069 | 1.236 | ± | 0.075 | F(2,92)= | 1.117 | 0.332 | 0.780 |
| MD | 1.572 | ± | 0.058 | 1.590 | ± | 0.061 | 1.598 | ± | 0.055 | F(2,92)= | 2.176 | 0.120 | 0.588 |
| FA | 0.797 | ± | 0.063 | 0.790 | ± | 0.055 | 0.787 | ± | 0.063 | F(2,92)= | 0.304 | 0.738 | 0.930 |

FA=Fractional Anisotropy. MD=Mean Diffusivity. AD=Axial Diffusivity. RD=Radial Diffusivity. MD, AD and RD measures were multiplied by 1000. Analyses were corrected for age and sex.

**Supplementary Table 8.** Group differences between patients and controls in the interaction between white matter measures and affective empathy subscale scores

|  | **group * white matter * affective empathy** | | | |
| --- | --- | --- | --- | --- |
|  | **F** | | **p (adj)** | **Cohens’ d** |
| *Personal distress* |  |  |  |  |
| anterior thalamic radiation AD | F(1,92)= | 5.89 | 0.017* | 0.51 |
| anterior thalamic radiation RD | F(1,92)= | 0.03 | 0.859 | 0.04 |
| anterior thalamic radiation MD | F(1,92)= | 1.13 | 0.291 | 0.22 |
| anterior thalamic radiation FA | F(1,92)= | 1.07 | 0.304 | 0.22 |
| *Empathic concern* |  |  |  |  |
| anterior thalamic radiation AD | F(1,92)= | 5.02 | 0.003* | 0.47 |
| anterior thalamic radiation RD | F(1,92)< | 0.01 | 0.958 | 0.01 |
| anterior thalamic radiation MD | F(1,92)= | 1.32 | 0.254 | 0.24 |
| anterior thalamic radiation FA | F(1,92)= | 1.03 | 0.312 | 0.21 |

Significance level p is adjusted for multiple comparison (FDR). Analyses were corrected for age, sex, and scanner.

**Supplementary Table 9.** Information on the association between affective empathy and measures of white matter diffusivity for (A) patients with schizophrenia and controls and for (B) patients with lower performance on emotion recognition (PT-low), patients with normal emotion recognition (PT-normal) and controls after adding IQ as additional covariate.

|  | *Means (sd)* | | | | | | *Statistics* | | |
| --- | --- | --- | --- | --- | --- | --- | --- | --- | --- |
|  | **Controls** | | **Patients** | | | | **F** | **p (Adj)** | **Cohen’s d** |
| 1. *Affective empathy* |  | |  | |  | |  |  |  |
| anterior thalamic radiation FA | 0.834 | (0.055) | 0.828 | | (0.040) | | F(1,92)=0.57 | 0.454 | 0.16 |
| anterior thalamic radiation RD | 1.113 | (0.080) | 1.119 | | (0.045) | | F(1,92)=0.01 | 0.912 | 0.02 |
| anterior thalamic radiation MD | 1.470 | (0.064) | 1.472 | | (0.040) | | F(1,92)=1.55 | 0.216 | 0.26 |
| anterior thalamic radiation AD | 2.182 | (0.056) | 2.179 | | (0.056) | | F(1,92)=5.25 | 0.024* | 0.48 |
| 1. *Interaction (affective empathy & emotion recognition)* |  | | **PT-normal** | | **PT-low** | |  |  |  |
| anterior thalamic radiation FA | 0.834 | (0.055) | 0.831 | (0.041) | 0.817 | (0.035) | F(2,92)=0.59 | 0.556 | 0.16 |
| anterior thalamic radiation RD | 1.113 | (0.080) | 1.116 | (0.047) | 1.129 | (0.038) | F(2,92)=0.94 | 0.935 | 0.06 |
| anterior thalamic radiation MD | 1.470 | (0.064) | 1.471 | (0.041) | 1.477 | (0.039) | F(2,92)=1.44 | 0.243 | 0.25 |
| anterior thalamic radiation AD | 2.182 | (0.056) | 2.181 | (0.054) | 2.174 | (0.062) | F(2,92)=3.55 | 0.033 | 0.39 |

Mean and SD of AD, RD and MD are multiplied x1000. Significance level p is adjusted for multiple comparison (FDR).

* significant at p(adj)<0.05

^a^ The direction of the interaction AD measure; HC> PT-normal>PT-low, respectively (p=0.047, p<0.001, p=0.011).

**R libraries;**

*Short list*

boot~1.3-20, car~2.1-6, corrplot~0.84, data.table~1.10.4-3, fdrtool~1.2.15, Formula~1.2-2, gdata~2.18.0, ggplot2~2.2.1, gridExtra~2.3, heplots~1.3-4, Hmisc~4.1-1, lattice~0.20-35, lme4~1.1-15, Matrix~1.2-12, moments~0.14, nlme~3.1-131, plyr~1.8.4, psy~1.1, QuantPsyc~1.5, rJava~0.9-9, survival~2.41-3, tableone~0.9.2, xtable~1.8-2, fdrtool~1.2.15.

*Detailed list:*

R Core Team (2017). _R: A Language and Environment for Statistical Computing_. R Foundation for Statistical Computing,Vienna, Austria. <URL: https://www.R-project.org/>.

Falissard B (2012). _psy: Various procedures used in psychometry_. R package version 1.1, <URL:

https://CRAN.R-project.org/package=psy>.

Urbanek S (2017). _rJava: Low-Level R to Java Interface_. R package version 0.9-9, <URL:

https://CRAN.R-project.org/package=rJava>.

Fletcher TD (2012). _QuantPsyc: Quantitative Psychology Tools_. R package version 1.5, <URL:

https://CRAN.R-project.org/package=QuantPsyc>.

Venables WN and Ripley BD (2002). _Modern Applied Statistics with S_, Fourth edition. Springer, New York. ISBN 0-387-95457-0, <URL: http://www.stats.ox.ac.uk/pub/MASS4>.

Canty A and Ripley BD (2017). _boot: Bootstrap R (S-Plus) Functions_. R package version 1.3-20.

Davison AC and Hinkley DV (1997). _Bootstrap Methods and Their Applications_. Cambridge University Press, Cambridge. ISBN 0-521-57391-2, <URL: http://statwww.epfl.ch/davison/BMA/>.

Komsta L and Novomestky F (2015). _moments: Moments, cumulants, skewness, kurtosis and related tests_. R package version 0.14, <URL: https://CRAN.R-project.org/package=moments>.

Klaus B and Strimmer. K (2015). _fdrtool: Estimation of (Local) False Discovery Rates and Higher Criticism_. R package version 1.2.15, <URL: https://CRAN.R-project.org/package=fdrtool>.

Wei T and Simko V (2017). _R package "corrplot": Visualization of a Correlation Matrix_. (Version 0.84), <URL: https://github.com/taiyun/corrplot>.

Bates D, Mächler M, Bolker B and Walker S (2015). "Fitting Linear Mixed-Effects Models Using lme4." _Journal of Statistical Software_, *67*(1), pp. 1-48. doi: 10.18637/jss.v067.i01 (URL: http://doi.org/10.18637/jss.v067.i01).

Bates D and Maechler M (2017). _Matrix: Sparse and Dense Matrix Classes and Methods_. R package version 1.2-12, <URL: https://CRAN.R-project.org/package=Matrix>.

Pinheiro J, Bates D, DebRoy S, Sarkar D and R Core Team (2017). _nlme: Linear and Nonlinear Mixed Effects Models_. Rpackage version 3.1-131, <URL: https://CRAN.R-project.org/package=nlme>.

Wickham H (2011). "The Split-Apply-Combine Strategy for Data Analysis." _Journal of Statistical Software_, *40*(1), pp. 1-29. <URL: http://www.jstatsoft.org/v40/i01/>.

Fox J, Friendly M and Monette G (2017). _heplots: Visualizing Tests in Multivariate Linear Models_. R package version 1.3-4, <URL: https://CRAN.R-project.org/package=heplots>.

Friendly M (2007). "HE plots for Multivariate General Linear Models." _Journal of Computational and Graphical Statistics_, *16*(4), pp. 421-444.

Friendly M (2010). "HE Plots for Repeated Measures Designs." _Journal of Statistical Software_, *37*(4), pp. 1-40. <URL:http://www.jstatsoft.org/v37/i04/>.

Fox J and Weisberg S (2011). _An R Companion to Applied Regression_, Second edition. Sage, Thousand Oaks CA. <URL:http://socserv.socsci.mcmaster.ca/jfox/Books/Companion>.

Warnes GR, Bolker B, Gorjanc G, Grothendieck G, Korosec A, Lumley T, MacQueen D, Magnusson A, Rogers J and others (2017)._gdata: Various R Programming Tools for Data Manipulation_. R package version 2.18.0, <URL:https://CRAN.R-project.org/package=gdata>.

Yoshida K and Bohn. J (2018). _tableone: Create 'Table 1' to Describe Baseline Characteristics_. R package version 0.9.2, <URL: https://CRAN.R-project.org/package=tableone>.

Auguie B (2017). _gridExtra: Miscellaneous Functions for "Grid" Graphics_. R package version 2.3, <URL:

https://CRAN.R-project.org/package=gridExtra>.

Dahl DB (2016). _xtable: Export Tables to LaTeX or HTML_. R package version 1.8-2, <URL:

https://CRAN.R-project.org/package=xtable>.

Dowle M and Srinivasan A (2017). _data.table: Extension of `data.frame`_. R package version 1.10.4-3, <URL:

https://CRAN.R-project.org/package=data.table>.

Harrell Jr FE, Dupont wcfC and others. m (2018). _Hmisc: Harrell Miscellaneous_. R package version 4.1-1, <URL: https://CRAN.R-project.org/package=Hmisc>.

Wickham H (2009). _ggplot2: Elegant Graphics for Data Analysis_. Springer-Verlag New York. ISBN 978-0-387-98140-6, <URL: http://ggplot2.org>.

Zeileis A and Croissant Y (2010). "Extended Model Formulas in R: Multiple Parts and Multiple Responses." _Journal of Statistical Software_, *34*(1), pp. 1-13. doi: 10.18637/jss.v034.i01 (URL: http://doi.org/10.18637/jss.v034.i01).

Therneau T (2015). _A Package for Survival Analysis in S_. version 2.38, <URL:

https://CRAN.R-project.org/package=survival>.

Terry M. Therneau and Patricia M. Grambsch (2000). _Modeling Survival Data: Extending the Cox Model_. Springer, New York. ISBN 0-387-98784-3.

Sarkar D (2008). _Lattice: Multivariate Data Visualization with R_. Springer, New York. ISBN 978-0-387-75968-5, <URL: <http://lmdvr.r-forge.r-project.org>>.

Bernd Klaus and Korbinian Strimmer. (2015). fdrtool: Estimation of (Local) False Discovery Rates and Higher

Criticism. R package version 1.2.15. https://CRAN.R-project.org/package=fdrtool
